# Supplementary material for: Progressive and Coordinated Mobilization of the Skeletal Muscle Niche throughout Tissue Repair Revealed by Single-Cell Proteomic Analysis
Source: Cells. 2021 Mar 28;10(4):744. doi: 10.3390/cells10040744 (PMC8066646; doi:10.3390/cells10040744)
Supplement: Supplementary file 1 [file cells-10-00744-s001.zip › cells-1092235 supp/cells-1092235 - Supplementary Fig 1.pdf]

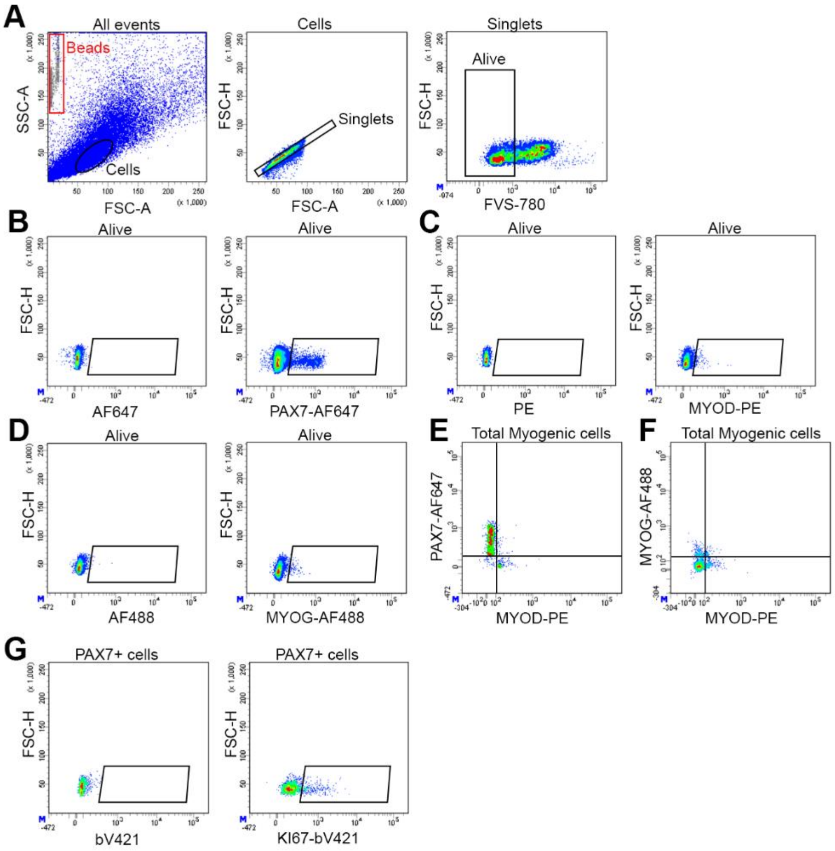

Supplementary Figure 1. Quantitative analysis of MuSC by flow cytometry. Mononucleated cells from single digested Tibialis anterior (TA) muscle were analyzed by flow cytometry at different time points post-injury, using Trucount™ Absolute Counting Tubes. (**A**) Debris, doublets and dead cells were excluded from the analysis. (**B-D**) Representative density scatterplots showing the expression of PAX7 (**B**), MYOD (**C**) and MYOG (**D**) at 7DPI. Gates were positioned on the basis of FMO controls for each marker (left panels). (**E-F**) Joined gate PAX7+ or MYOD+ or MYOG+ cells was considered as the Total Myogenic cells (100%). Repartition of PAX7/MYOD (**E**) and MYOG/MYOD (**F**) expression. (**G**) Proportion of PAX7+ cells expressing KI67 at 7DPI.
