# Supplementary material for: Progressive and Coordinated Mobilization of the Skeletal Muscle Niche throughout Tissue Repair Revealed by Single-Cell Proteomic Analysis
Source: Cells. 2021 Mar 28;10(4):744. doi: 10.3390/cells10040744 (PMC8066646; doi:10.3390/cells10040744)
Supplement: Supplementary file 1 [file cells-10-00744-s001.zip › cells-1092235 supp/cells-1092235 - Supplementary Fig 2.pdf]

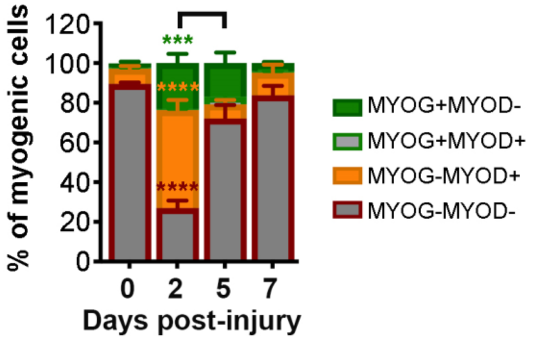

Supplementary Figure 2. Quantitative FACS analysis of mononucleated cells from single TAs. Graph showing the proportions of myogenic cells expressing MYOD and MYOG. Data are represented as the mean±sem of minimum 4 isolated TAs. One way ANOVA with ***$p < 0.0001$ and ****$p < 0.05$.
